# Supplementary material for: The genetic architecture of repeated local adaptation to climate in distantly related plants
Source: Nat Ecol Evol. 2024 Aug 26;8(10):1933–47. doi: 10.1038/s41559-024-02514-5 (PMC11461274; doi:10.1038/s41559-024-02514-5)
Supplement: Supplementary file 1 — Supplementary Methods 1–7, Results 1–7 and Figs. 1–12. [file 41559_2024_2514_MOESM1_ESM.pdf]

nature ecology & evolution

Supplementary information https://doi.org/10.1038/s41559-024-02514-5

The genetic architecture of repeated local adaptation to climate in distantly related plants

In the format provided by the authors and unedited

The genetic architecture of repeated local adaptation to climate in distantly-related plants - Supplementary Materials

Supplementary Methods:

1. Justification of repeatability threshold (FDR < 0.5)
2. Comparing the OrthoFinder tree against the proposed species tree
3. Covariance among climate variables
4. Justification of GEA methodology
5. Correcting for the effect of SNP count on Z_{WZA} heteroscedasticity
6. Alternative methods for deriving Orthogroup-level pleiotropy estimates
7. Randomisations to test the effect of Orthogroup structure

Supplementary Results:

1. Orthogroups tested for repeatability are representative of general genes in GEA analyses
2. Relative Niche-Breadth and GEA power do not predict contributions to repeatability
3. RAOs are not enriched for phylogenetic signal of GEA results
4. Orthogroups tested for repeatability may exhibit more repeatability than those not tested
5. Reduced sampling of the global species range may reduce contributions to repeatability
6. Gene duplication may facilitate adaptive repeatability
7. Recombination does not drive signals of GEA or repeatability

Supplementary Figures:

- Supplementary Figure 1: Summary of bioinformatics workflows
- Supplementary Figure 2: Associations between GEA power and species contributions to repeatability.
- Supplementary Figure 3: Associations between Niche Breadth (global mean) and species contributions to repeatability.
- Supplementary Figure 4: Associations between Niche Breadth (local mean) and species contributions to repeatability.
- Supplementary Figure 5: Brassicacae PicMin analysis of tested vs untested orthogroups.
- Supplementary Figure 6: Summary of orthogroup occupancy.
- Supplementary Figure 7: Agreement between TimeTree phylogeny and OrthoFinder phylogeny.
- Supplementary Figure 8: Expected PicMin p-value distributions under the null.
- Supplementary Figure 9: Overlap of RAOs across climate variables.

- Supplementary Figure 10: Demonstration of expected false-positives based on the FDR.
- Supplementary Figure 11: GEA ep-value distributions in tested vs untested orthogroups.
- Supplementary Figure 12: Associations between GEA ep-values and recombination rate.

Supplementary Tables:

- Supplementary Table 1: Original dataset details
- Supplementary Table 2: Orthogroup summary statistics
- Supplementary Table 3: Full PicMin statistical test results for RAOs
- Supplementary Table 4: Summary of species contributions across RAOs
- Supplementary Table 5: GO terms for RAOs
- Supplementary Table 6: *Arabidopsis thaliana* gene details in RAOs
- Supplementary Table 7: GO enrichment test results
- Supplementary Table 8: Estimates of pleiotropy for all tested orthogroups

SUPPLEMENTARY METHODS

1) Justification of repeatability threshold (FDR < 0.5)

The decision to categorise RAOs on the basis of an FDR-threshold of 0.5 was taken to include as many true-positives as possible in downstream functional enrichment analyses, while limiting the inclusion of false positives so that they do not make up the majority in our final set of RAOs. At this threshold, each RAO is at least as, or more, likely to be a true-positive than it is a false-positive. It is important to note that unlike p-values, FDR-adjusted p-value (q-value) thresholds do not carry any expectation for an expected number of q-values below the threshold, given the number of tests performed when all null hypotheses are true. For example, when performing 100, 1000 or 10,000 tests, a p-value threshold of 0.05 carries an expectation of approximately 5, 50, and 500 (5%) 'significant' tests below the threshold when all null hypotheses are true. In contrast, a q-value threshold of 0.5 carries an expectation of ~1 test being significant below the threshold (with a 50% chance of being a false-positive) regardless of the number of tests performed when all null hypotheses are true (Fig S9). This is because the FDR-threshold explicitly defines the proportion of results below that threshold that are expected to be false-positives, not the absolute number of false-positives. Consequently, an enrichment of tests with q-values <0.5 relative to null expectations can be considered a demonstration of an enrichment of true-positives. Put another way, an enrichment of tests with q-values <0.5 is an indication of a non-uniform p-value distribution that is weighted towards more lower values.

We tested this by randomly shuffling ep-values$_{WZA}$ within species within each climate variable 1000 times. We then performed all PicMin tests as for our observed data, FDR-adjusted the p-values within each climate variable, and summed together the number of orthogroups with an FDR q-value <0.5 across the 21 climate variables. Across the 1,000 permutations, we observe an expected median of 36 (mean = 37.9) RAOs with an FDR of <0.5 (between 1 and 2 per climate variable, per randomisation, in line with random uniform expectations) and a maximum of 102. Similar expected values (median = 36, mean = 37.8, max = 102) were obtained when reducing RAOs to unique orthogroups (i.e. an orthogroup may be significant across multiple climate variables). In our observed data we detected 141 RAOs, or 108 unique RAOs, representing a significant enrichment when compared to null expectations.

2) Comparing the OrthoFinder tree against the proposed species tree

The topology among major clades of the inferred species tree among reference genome species had good agreement with the equivalent species tree from TimeTree (Fig S7), although there were disagreements regarding sister taxa at the ancestral node of the Eudicot species (all species excluding *Pinus taeda, Picea abies* and *Panicum hallii*) and within *Populus* and Brassicacae clades. Our species tree's grouping of *P. trichocarpa* and *P.*

deltoides as sisters is supported by phylogenomics work in *Populus*[1], and our grouping of *Capsella rubella* and *Boechera stricta* as sisters within Brassicaceae is supported by additional phylogenomics[2].

3) Covariance among climate variables

The 19 bioclim variables studied here are, of course, not independent of one another and exhibit substantial covariance. This is to be expected given various bioclim variables are calculated from other bioclim variables, for e.g. isothermality (BIO3) = 100 * mean diurnal range (BIO2)/ temperature annual range (BIO7). In other cases, bioclim variables are simply similar observations, for e.g., maximum temperature in the warmest month (BIO5) and mean temperature in the warmest quarter (BIO10). Many studies seek to remove non-independence within environmental datasets through reducing dimensionality with principal component analysis (PCA) or redundancy analysis (RDA). Reducing dimensionality, whilst reducing nonindependence, does however add significant complications in terms of interpretation, where it can be unclear which environmental contributors to a combined variable are responsible for adaptation. Additionally, such approaches are undesirable in studies of multiple datasets where covariance of bioclim variables will vary among individual datasets, such that combined variables are incomparable. One approach to this issue may be to include environmental data from all datasets in a single dimensionality reduction, however this approach tends to maximise the significance of variation among datasets, which greatly exceeds variation within datasets, and is substantially less relevant to selection pressures and adaptation within datasets. Consequently, the simplest approach in terms of downstream interpretation and making comparisons across datasets is to maintain individual variables and acknowledge the potential non-independence of results among likely covarying variables (Fig S10).

Our climate change variables did exhibit some association with other bioclim variables, particularly those linked with seasonality (Extended Data 6). This is likely due to the increased variance of monthly max temperature and precipitation at sites with greater seasonality, which will dampen effect size estimates between decades.

4) Justification of GEA methodology

We performed GEAs using Kendall's τ correlations that do not correct for population structure for several reasons. Firstly, the non-parametric correlation makes no assumptions about the distribution of allele frequency or environmental variation which is likely to vary substantially among datasets and climate variables. The Kendall's τ correlation is a rank-based correlation similar to Spearman's ρ but with adjustments to handle ties. There are an abundance of approaches to perform GEA, some of the most popular being BayPass[3], PCAdapt[4], latent-factor mixed modelling (LFMM)[5], and RDA[6]. Each of these approaches includes a correction for potentially spurious associations between allele frequencies and environment driven by spatial autocorrelation between population structure/gene flow and

environmental variation. In doing so, these approaches should theoretically reduce false-positives (Type-I error) but incur a drop in power (more false-negatives, Type-II error)[7,8], as seen empirically in other studies[9,10]. However, a recent study deploying these methods on datasets created using individual-based simulations of evolution found substantial variation in realised power and false positive rates, with LFMM exhibiting a high power but a false positive rate near 100%, while RDA showed both very low power and low false positive rate[11]. Low power for methods including population structure correction was also observed by another simulation study assessing the accuracy of GEA methods[12]. Thus, methods that attempt to "control for population structure" cannot reliably disentangle the contributions of genetic drift and natural selection to observed patterns of allele frequency variation within a single species when axes of environmental variation align with axes of population structure. For studying climate variation, which is inherently spatial, axes of climatic variation are expected to align with spatial axes of population structure. This is especially the case in real empirical datasets where complex phenomena like allele surfing might be particularly problematic[13].

In contrast, our method avoids the pitfalls of correction for population structure by instead relying on comparisons across species to more reliably detect the signature of natural selection repeatedly driving local adaptation. This works better than single-species analyses because if random genetic drift is driving evolution at a given gene in multiple species, we can specify the probability of repeatedly observing a strong association by chance and perform our tests accordingly. There is therefore no need to correct for population structure at the level of the within-species analysis, because this is controlled by our among-species probability model, as deployed in PicMin.

It should be noted however that some major historical expansion and contraction events are shared across species and may have facilitated admixture among closely-related species, for example in Northern Eurasia following glaciation-interglaciation cycles[14]. To rule out the potential for such introgression to be driving our observations, we visualised the shared contributions of closely-related species and general phylogenetic signal within our repeatability results and statistically tested phylogenetic signal in our repeatability results.

5) Correcting for the effect of SNP count on Z_{WZA} heteroscedasticity

The uncorrected Z_{WZA} exhibits heteroscedasticity associated with increased Z_{WZA} variance in genes with more SNPs. We took several post-processing steps to account for this. Firstly, for the most SNP dense genes, we down sampled SNPs in genes with SNP counts above the 75% quantile to the 75% quantile, taking the per gene Z_{WZA} score as the mean Z_{WZA} calculated over 100 down sampled SNP sets. We also trimmed genes with minimal SNP information, removing genes with fewer than 5 SNPs, or if the 5% quantile of per gene SNP count was less than 5, we removed the bottom 5% of genes based on SNP count. This process therefore removed, at most, the bottom 5% of genes. We took a modified approach to the

WZA for standardising for SNP count across genes. Because the WZA is an approach designed to detect local adaptation, the WZA conservatively estimates the expected mean and variance of Z_{WZA} for a gene of given SNP count by approximating the relationship between Z_{WZA} mean and variance and SNP count using all observed data. However, true-positive GEA outliers could upwardly bias estimates of Z_{WZA} variance under this approach. Because we do not expect false-positive outliers to appear repeatedly across species, we adopted a less conservative approach and only estimated Z_{WZA} mean and variance from the lower half of Z_{WZA} scores ($Z_{WZA} < 0$). For our analysis, this should improve power within species at the cost of potentially identifying more false-positives, but these should be removed later in the across-species repeatability analysis. Having an approximation for the expected mean and variance of Z_{WZA} for a gene with a given number of SNPs, we calculated a parametric p-value for each gene on the basis of the observed Z_{WZA} using the *pnorm()* function.

6) Alternative methods for deriving Orthogroup-level pleiotropy estimates

To condense our per gene estimates of tissue specificity to per orthogroup estimates, we initially approached this in two ways. We transformed τ scores into per gene ep-values based on rank, treating either higher τ estimates (and higher specificity) as lower ep-values or lower τ values (and higher pleiotropy) as lower ep-values. We explored both of these approaches to ensure that choice of the most specific vs. least specific paralog per orthogroup did not affect the interpretation of pleiotropy drawn from this analysis. In each case, we then retained the minimum ep-value per orthogroup and corrected for paralogs with a Dunn-Šidák correction. Finally, we transformed per orthogroup ep-values to *Z*-scores (Z_{τ}) with a mean of 0 and sd of 1 across all orthogroups.

This approach therefore reflects our approach based on Tippett's method for condensing per gene GEA results to orthogroups. This approach was preferred over taking the mean τ per Orthogroup as there is no assumption that paralogs should retain specificity/pleiotropy. Indeed, taking the mean τ per orthogroup greatly reduced the occurrence of high τ values in the genome-wide distribution suggesting paralogs within orthogroups vary in their specificity of expression, which may occur due to neofunctionilisation or subfunctionalisation[15]. Whilst we observed that the number of paralogs decreases as evidence for repeatability increases (Extended Data 8B), it is important to note that this is not a feature introduced by the data structure and having to correct for the number of paralogs (Extended Data 8B and Fig 5E).

7) Randomisations to test the effect of Orthogroup structure

A potential bias between evidence for repeatability and duplication metrics could be introduced due to our method for processing per orthogroup ep-values requiring a Dunn-Šidák correction based on the number of paralogs (reducing the statistical power more in bigger orthogroups). To test this, we took the observed per gene ep-values associated with

all climate variables and shuffled them 100 times within species and within climate variables before correcting for the number of paralogs as normal. In total this creates 2,100 randomised single-variable p-value sets (21 variables * 100 randomisations). This approach therefore shuffles the biological information contained within the per gene ep-values but retains the orthogroup and paralog structure within the dataset across species and climate variables. We then ran PicMin on each of the 2,100 sets of randomised data and within each of the 100 randomised sets, calculated the minimum PicMin p-value across the 21 randomised climate variables and used these to group orthogroups into deciles as for our observed data. We could then calculate the mean duplication metrics per decile as for our observed data, and repeated this 100 times to derive 100 randomised means per decile to compare against our observed decile means.

SUPPLEMENTARY RESULTS

1) Orthogroups tested for repeatability are representative of general genes in GEA analyses

A concern when testing for repeatability across species is whether or not the genes within orthogroups that are actually tested for repeatability are representative of all genes. Only genes that were in orthogroups with at least 20 species represented and with no fewer than 10 paralogs were tested for repeatability with PicMin. This decision was made to reduce the total number of tests performed by only testing orthogroups with the most data and so greatest power to detect repeatability across diverse species. The genes in these tested orthogroups (N = 8,470) represented varying proportions of the total genes tested by GEA per species, ranging from 37.1% in *Picea abies* to 61.6% in *Panicum hallii* (Table S2). We first plotted the distributions of per orthogroup ep-values in tested vs not-tested orthogroups. These plots, summarised for precipitation in the dry month, demonstrate that in general the distribution of tested ep-values for a given climate variable were approximately uniform across most species and did not differ substantially from those that were not tested in most cases (Fig S11). If anything, orthogroups tested for repeatability were more likely to include probable adaptive genes than orthogroups that were not tested for repeatability, evidenced as inflated densities of lower per gene GEA ep-values in tested vs untested distributions.

2) Relative Niche-Breadth and GEA power do not predict contributions to repeatability

The variability with which species contribute towards signatures of repeatability across climate variables (Fig 3A) begs the question of whether there are features of individual GEA (individual here referring to any given pair of species-climate) that explain this variation. The focus on individual species-climate tests, as opposed to the overall total number of RAOs, sets this question apart from those in the section “Reduced sampling of the global species range may reduce contributions to repeatability”. Variability of contribution could be explained by the power attached to each individual GEA, with the assumption being that species contribute more towards repeatability for GEA with greater power as a result of lower Type-II error. To examine this, we looked at two features of GEA that are likely to affect power: 1) the proportion of genetic variance that can be explained by climatic variation (hereafter GSEA - Genetic Structure Environment Association); 2) the relative niche-breadth (NB) of climatic variation, which may reflect strength of selection across the sampled range. We note that in contrast to the LOO analysis above, here, niche breadth is calculated in two different ways: one that represents the breadth relative to other species and another that accounts for scaling between the breadth measure and magnitude of the mean. As opposed to approximating the proportion of the global climate niche that has been sampled within each dataset, our measures of niche breadth here attempt to approximate comparable estimates of climate variability among species. For example, here

we are quantifying whether the sampling of species X experiences a greater variability of a given climate variable than the sampling of species Y, as opposed to measuring the extent to which the sampling of species X covers species X's global climate niche.

We calculated GSEA using partial redundancy analysis (pRDA). We constructed models to explain the variance in a response matrix of per site allele frequencies, randomly sampled to 10,000 SNPs (with at least 90% of non-missing data per SNP), by predictor matrices of climate variation and spatial variation (latitude and longitude). We opted for latitude and longitude representations of space because these capture the assumptions of isolation-by-distance and spatial autocorrelations with the environment. Alternative approaches, such as Moran's spatial eigenvectors, accurately capture spatial information in terms of clustering of populations, but it is difficult to interpret these alongside how environmental variation may similarly covary with space. We produced a separate model for each bioclim variable individually, as opposed to modelling all together, in order to link genetic variance explained by individual climate variables back to individual variable GEAs. We considered the total proportion of genetic variance explained by climate as the combined partitions of genetic variation that could be explained exclusively by climate and that could be explained by either climate or space. If a greater proportion of neutral population structure is aligned with axes of environmental variation, GEA may exhibit reduced power as a result of truly adaptive genes co-segregating with an appreciable proportion of genome-wide neutral genetic variation[11]. We predicted that within species, the strongest contributions towards repeatability signatures would be observed when GSEA was lowest. Across species, however, we did not observe any consistent relationship between GSEA and contribution towards signatures of repeatability (Fig S2).

We calculated niche breadth in two ways to account for variability in means and distribution shapes across variables and species. Firstly, for each dataset and each climate variable, we calculated the species range (max - min) of climatic variation, and then standardised this based on the global range within climate variables across all species from locations with genome-sequenced samples included (i.e. not from GBIF). This estimate of niche breadth therefore captures the proportion of global variation present within an individual dataset, with the prediction being that the datasets contributing most to repeatability for a given climate variable will be those where this proportion is greatest. Secondly, we estimated niche-breadth by standardising species' range values by the mean climate value within each range. This estimate of niche breadth therefore contextualises previous estimates as a proportion of their mean. We opted to explore both of these estimates of niche breadth due to subtle differences in interpretation that come with standardising by either the global range or local mean. Take for example two ranges of precipitation variation between 100-200 mm and 3000-3100 mm. Each of these ranges is equivalent if standardised by the global range, however we expect that a 100mm difference in rainfall may be more significant if it represents a doubling in annual precipitation relative to a small increase. Standardising by

the local mean therefore captures differences in relative niche breadth. In each case, we observe no evidence that niche breadth variation is associated with contributions towards signatures of repeatability (Fig S3-S4).

3) RAOs are not enriched for phylogenetic signal of GEA results

It is expected that species that are more closely-related may contribute disproportionately to signatures of repeatability, particularly if closely-related species share adaptive variation. Consequently, clusters of closely-related species, for example our Brassicaceae species or *Helianthus* or *Eucalyptus* species groups, may be disproportionate contributors to repeatability in our dataset. On the other hand, the PicMin test assumes that each species is independently evolving, so shared standing variation among closely-related species could introduce non-independence into the test. Thus, it is important to assess whether our results include a signal of closely-related species having increased repeatability. A visual inspection of contributions towards repeatability suggests that this is not the case (Fig 3B, Extended Data 3). However, we also tested each RAO for phylogenetic signal in the distribution of GEA ep-values among species tips. To do this, we took each of the 141 RAOs (some of the 108 unique RAOs were represented across multiple climate variables) and used the *phytools::phylosig()*[16] function to test each orthogroup for phylogenetic signal of -log10-transformed GEA p-values against random expectations under Brownian evolution. We used the 'K' method [17] with 1,000 sims (we also used the 'lambda' method and obtained the same result). To derive a species-level phylogenetic tree with branch lengths (Extended Data 2A), we curated our reference genome tree (Fig 1D) and where multiple species were mapped to the same reference genome (for example *Helianthus* and *Eucalyptus*) we split tips to include all species and separated species by the minimum branch length in the original reference genome phylogeny. We then asked whether the average 'K' value observed in the 141 RAOs differed from 1,000 random draws of 141 orthogroups (excluding the 141 RAOs).

There was limited evidence of significant phylogenetic signal of GEA ep-values within the 141 RAOs. Seven RAOs had phylogenetic clustering signal p-values <0.05, in line with null expectations (5% of tests). The mean 'K' value of observed RAOs was 0.081, which was actually lower than the mean 'K' (0.086) of 1,000 randomly chosen groups of orthogroups (Extended Data 2B). These results demonstrate that there was limited evidence of phylogenetic signal driving signatures of repeatability within RAOs, and RAOs did not exhibit elevated phylogenetic signal relative to orthogroups without evidence of adaptive repeatability.

4) Orthogroups tested for repeatability may exhibit more repeatability than those not tested

It is expected that repeatability should decline with increasing TMRCA between species. This is due to several factors, including reduced likelihood of sharing common adaptive variants,

functional divergence of genes, and changes to species general biology and physiology[18]. We observed no evidence of phylogenetic signal in our RAOs, however. One explanation for this may be that the orthogroups tested for repeatability are reasonably conserved, potentially negating the expected trend at the genome-level of reduced repeatability through increased functional divergence of genes. To examine this, we repeated the PicMin repeatability analyses over a set of orthogroups found in the 7 Brassicaceae species in our dataset.

This set of orthogroups comprised 6,914 orthogroups in total. Of these, 5,620 were tested in the original analysis ('Full Dataset'), and 1,294 were tested here for the first time ('Brassica Dataset'). Of the 1,294 newly tested orthogroups, 374 orthogroups contained genes that were only found in Brassicaceae genomes ('Brassica Unique'). We repeated PicMin analyses using the same method as for the main analysis, and tested repeatability for orthogroups with all seven species.

In total, 25 orthogroups exhibited evidence of repeatability at our FDR <0.5 threshold, all of which were orthogroups previously tested in our main analysis. In addition to this, visualising the distribution of PicMin p-values across all climate variables by orthogroup status highlighted that orthogroups that had been tested in our main analysis exhibited stronger evidence of repeatability in this analysis (Fig S5). This result was consistent if orthogroups were grouped by previously-tested vs newly-tested, or unique to Brassicaceae vs not unique. In this re-analysis, all orthogroups had equal statistical power (7 species), and so this variability cannot be explained by variable power in orthogroups that were not tested previously. These results lend support to the idea that orthogroups with higher levels of conservation across species (i.e. those that contained enough species to be tested in our main analysis) may be more likely to be repeatedly involved in adaptation. A potential mechanism for this might be a reduction in functional divergence that is expected with increased conservation across diverse species.

5) Reduced sampling of the global species range may reduce contributions to repeatability

To address the potential of sampling bias in the repeatability analyses, and to explore variability among species in contributing towards repeatability, we quantified a range of features related to dataset quality. To understand how these features may affect the repeatability analyses, we used a Leave-One-Out (LOO) cross-validation approach that involved removing each species from the overall PicMin analysis and repeating the PicMin process, whilst accounting for the N-1 species. This LOO procedure produced a species-level value reflecting the increase or decrease in the number of FDR <0.5 orthogroups detected, which we then compared against our dataset quality features.

Dataset quality features were selected to reflect either biological or technical limitations of datasets and included the following: 1) Approximate climate extent covered by sequenced

dataset, relative to total climatic range of the species; 2) approximate geographic extent covered by the dataset, relative to global range; 3) percentage of total genes within the genome covered by sequencing data; 4) number of SNPs (averaged through datasets within species) per species; 5) number of individuals sampled (averaged through datasets within species) per species; 6) number of sampling locations (averaged through datasets within species) per species; 7) ratio of the number of individuals to number of sampling locations.

Features 1) and 2) were approximated by pulling all occurrence data for each species from GBIF (GBIF Occurrence Download https://doi.org/10.15468/dl.mcbger Accessed from R via *rgbif* [https://github.com/ropensci/rgbif] on 2024-05-03). For 1), the climatic niche of each species was calculated by extracting bioclim variables for all locations in GBIF and reducing dimensionality using scaled principal components analysis (PCA). The climate values for the sampled ranges were then projected onto the species-specific climate PCA. To compare the sampled niche to the global niche for each species, we calculated hypervolume overlap over each PC using dynamic range boxes (dynRB_Pn function in the dynRB package[19]). Overall hypervolume overlap was then calculated as the weighted mean overlap across individual principal components, where weights reflected the relevant eigenvalues, i.e. overlap on PC1 is more significant than PC10. For 2), we used the same occurrence data to calculate the average distance (in km) between a given pair of species occurrence points. Where species had >1000 occurrences in GBIF, we took 100 random subsets of 1,000 occurrences and performed pairwise distance calculations within subsets, keeping only intracontinental distances to avoid including distances across oceans. For our sampled data points, we took the same approach and calculated the mean distance (in km) between all paired locations. We then calculated the ratio of the average sampled distance to the average global distances. Whilst this measure is crude, it should capture the main information we are interested in here, i.e. distinguishing between a species where we have data from across the majority of its known geographic distribution as opposed to a small minority. Feature 3) was calculated on the basis of the number of genes with Z_{WZA} scores relative to the number of genes in the total OrthoFinder2 outputs for each genome. Features 4-7 were extracted from each of the original VCF files, and are found in Table S1. Features 1 and 2 reflect biological quality, in terms of the extent of biological variation sampled within our datasets. The remaining features reflect technical quality related to the sequencing of samples and statistical power.

Removing individual species had a variable effect on the retained number of PicMin RAOs (FDR <0.5 & <0.3, Extended Data 4A-B). The largest increase in the number of RAOs (relative to the total dataset count of 141 RAOs) was observed when removing *Amaranthus tuberculatus* (211 FDR <0.5 RAOs), whereas the largest reduction in RAOs was observed when removing either *Eucalpytus albens* or *E. sideroxylon* (89 FDR <0.5 RAOs). Interestingly, despite the removal of either of these *Eucalyptus* resulting in the same number of retained RAOs, the RAOs that were removed from the full set were generally not the same.

Specifically, of the RAOs removed from the original 141 by removing each of *E. albens* and *E. sideroxlyon*, only 50.6% were shared between the two eucalypts. This result is in agreement with the results presented in Fig 3B and Extended Data 2, suggesting a negligible influence of common or shared contributions towards repeatability between these species, or any closely-related species. In general, the change in the number of RAOs compared to when including all species reflected a mix of original RAOs dropping out and previously undetected RAOs becoming more significant. To give an example, of the 89 RAOs observed when excluding *E. sideroxlyon*, 74 of these were retained in the original 141 and 15 were newly detected.

When exploring the effect of dataset features on the variability of the LOO results among species (Extended Data 4C), the strongest associations were negative associations between the change in FDR <0.5 RAOs (LOO CV Change) and geographic sampling extent (Global Range Distance Ratio, $\rho = -0.43$) and climate niche sampling breadth (Global Climate Niche Overlap, $\rho = -0.33$) (Extended Data 4D). This highlights that the main feature of datasets underlying the potential power of each species in our repeatability analysis is biological, i.e. the extent of global species-level biological variation that has been sampled. Specifically, it suggests that removing species where less of the natural variation has been sampled tends to increase the number of RAOs, and vice-versa.

6) Gene duplication may facilitate adaptive repeatability

Gene duplication has been invoked to explain repeatability variation, notably among conifer species[20]. Duplications may facilitate repeatable evolution by alleviating functional constraints through sub- or neofunctionalisation[15]. We asked whether RAOs differed for the total number of duplications within each gene tree, the number of species-specific duplications (where all nodes downstream of the duplication involve a single genome), the number of single-copy genes within each gene tree, and whether species contributing towards repeatability within orthogroups were enriched for duplications (Extended Data 8A).

Our per orthogroup pleiotropy metrics were negatively associated with the number of gene duplication events per orthogroup (expression breadth: $\rho = -0.328$, $p < 2.2^{-16}$; node degree: $\rho = -0.383$, $p < 2.2e^{-16}$; Fig 5E). Importantly, however, randomising per gene pleiotropy scores did not produce any association with the number of duplication events per orthogroup, demonstrating that the orthogroup structure and analysis is not expected to produce spurious associations.

As observed for pleiotropy, grouping orthogroups by their strongest evidence of repeatability highlighted a clear tendency for gene duplication to vary with evidence of adaptive repeatability. Orthogroups with stronger evidence of repeatability were characterised by fewer duplications, fewer species-specific duplications, and a greater

number of single-copy genes (Extended Data 8B). Importantly, these associations with gene duplication were not observed following randomisation within species of per gene GEA ep-values (black bars in Extended Data 8B). Species contributing towards repeatability in RAOs (PicMin FDR < 0.5) did not differ in terms of per species duplications from species with low GEA ep-values (<0.1) from randomly drawn orthogroups (10,000 permutations, $p = 0.127$). These results therefore suggest that orthogroups with reduced gene duplication may be more likely to be repeatedly associated with adaptation, however it may be difficult to separate out the effects of pleiotropy and duplication, given duplication may promote subfunctionilisation and specialism among duplicated genes[21]. Indeed, per orthogroup duplication events were negatively associated with per orthogroup pleiotropy metrics (Fig 5E).

Interestingly, however, species that were contributing towards repeatability within RAOs were less likely to be single-copy genes within RAOs with respect to random expectations derived from taking species with low GEA ep-values within random sets of orthogroups (Extended Data 7C). This result therefore contradicts the notion that repeatedly adaptive orthogroups may be associated with reduced duplication, and is in line with previous observations in conifers[20]. The discrepancy between these results may be explainable due to noisy per orthogroup estimates as opposed to potentially more relevant per species estimates of duplication.

7) Recombination does not drive signals of GEA or repeatability

Recombination rate landscapes, potentially shared to some extent among closely-related species, represent a potential source of bias within our analyses due to the risk that WZA variance may be greatest in regions of low recombination[22]. It is unlikely that this source of bias has a strong effect on our repeatability results, given repeatability is observed to vary among climate variables, all of which are expected to be influenced in the same way by the recombination landscapes within species. Still, we wanted to quantify its effect here for a subset of datasets for which recombination rates were available. Recombination rates were acquired for the following genomes: *Arabis alpina*[23], *Arabidopsis lyrata*[24], *Arabidopsis thaliana*[25] and *Helianthus annuus*[26]. These corresponded to nine individual datasets. To examine associations between recombination rate and GEA results (WZA scores corrected for SNP count), we plotted GEA ep-values against ep-values estimated for individual genes according to the weighted-mean recombination rate over a given gene. We plotted four random climate variables as a demonstration (Fig S12).

Across the Brassicaceae, there is no observable association between recombination rate and GEA. Each distribution plotted is a uniform distribution of empirical p-values, thus the expected plot when association is minimal reflects regions of density across the whole plotting space. The association is complicated for the *Helianthus* species, however, exhibiting contrasting associations. *H. annuus*, and to a lesser extent *H. argophyllus*, exhibit

an association between GEA variance and low recombination, which can be seen particularly clearly for max temp warmest month and *H. annuus* as all high and low GEA ep-values are observed in regions of low recombination (low recombination ep-value). Conversely, *H. petiolaris* exhibits a linear association whereby GEA p-values are generally lower in regions of low recombination. This discrepancy between the Brassicaceae and *Helianthus* may reflect the known adaptive significance of regions of low recombination in these data from *Helianthus spp.*[27], therefore these associations may be genuine and biological. In support of this, the association between GEA results and recombination appear stronger in accordance with the proportion of each species genome where large non-recombining regions are observed in the original study (*H. argophyllus* has the lowest proportion of the genome covered, *H. petiolaris* has the most). Importantly, given that the effect of recombination is not observed across all datasets, it is unlikely to bias our estimates of repeatability. In addition, even though *Helianthus spp.* do exhibit an association, they do not show any evidence of contributing excessively to our estimates of repeatability in Fig 3A-B.

We also looked into whether genes within the same orthogroup were repeatedly associated with low recombination across species, and whether these overlapped significantly with our RAOs identified across GEA. To do this, we looked at the recombination rates from each of the four species and condensed the ep-values to per orthogroup p-values using the same Tippett's approach as was used for the GEA data. We then tested the same 8,470 orthogroups that were tested for climate data using PicMin, in order to identify orthogroups with repeatedly low recombination across the four species. We also removed orthogroups that did not have recombination estimates in all four species, leaving 7,446 to test with PicMin. This analysis identified 9 orthogroups with evidence of repeatedly low recombination across species at an FDR < 0.5. Of these 9, 1 was also identified as an RAO associated with climate at FDR < 0.5 (N = 108). An intersection of 1 does represent an almost ten-fold enrichment above the random expectation of 0.11, however it also only represents >1% of the identified RAOs associated with climate adaptation. From this, we can conclude that some repeatability identified through our GEA analyses may be driven by common regions of low recombination, however the extent of this effect is likely minimal.

SUPPLEMENTARY FIGURES

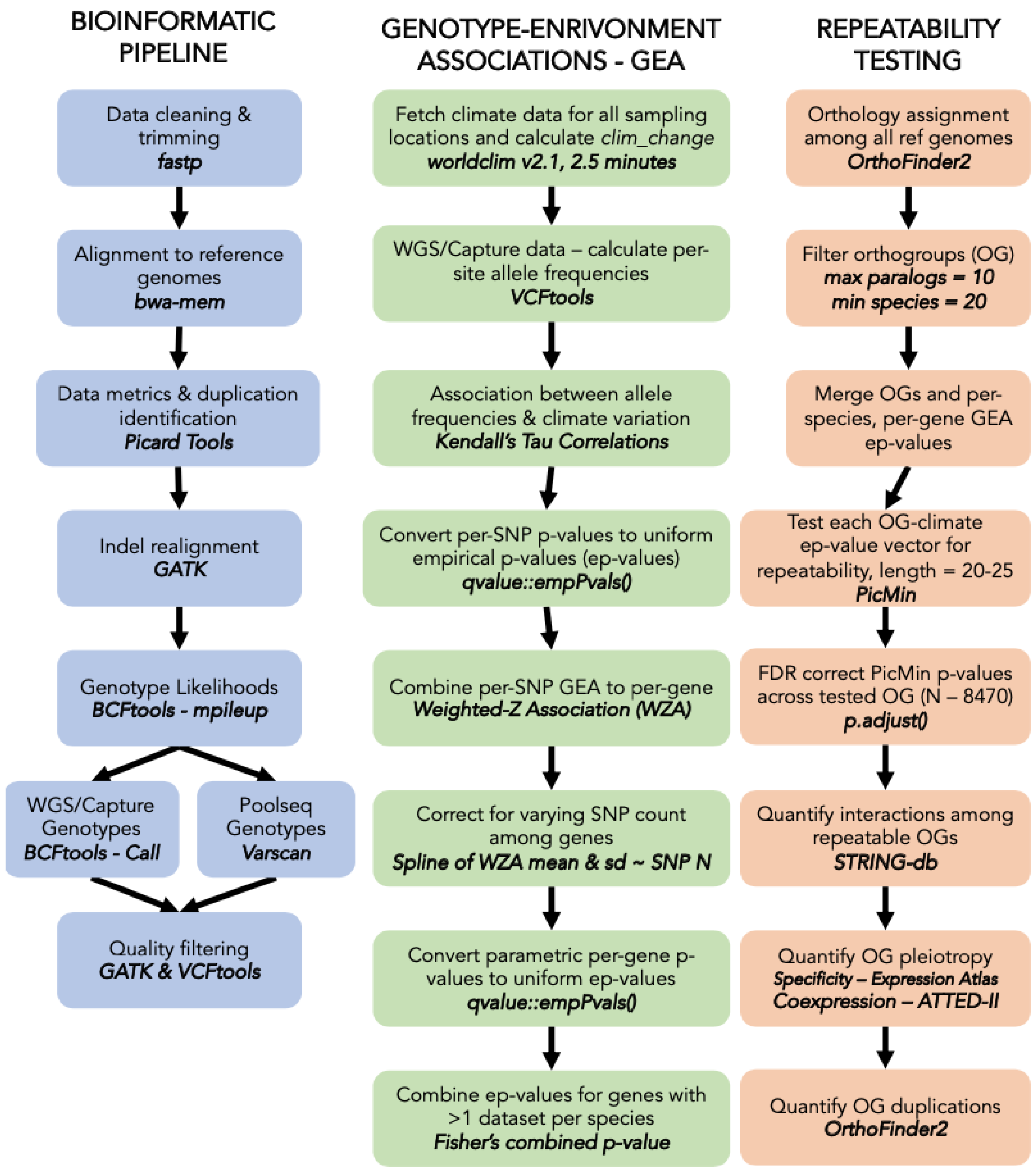

Supplementary Figure 1: *Summary of workflows for SNP-calling, GEA and testing for repeatability signatures.*

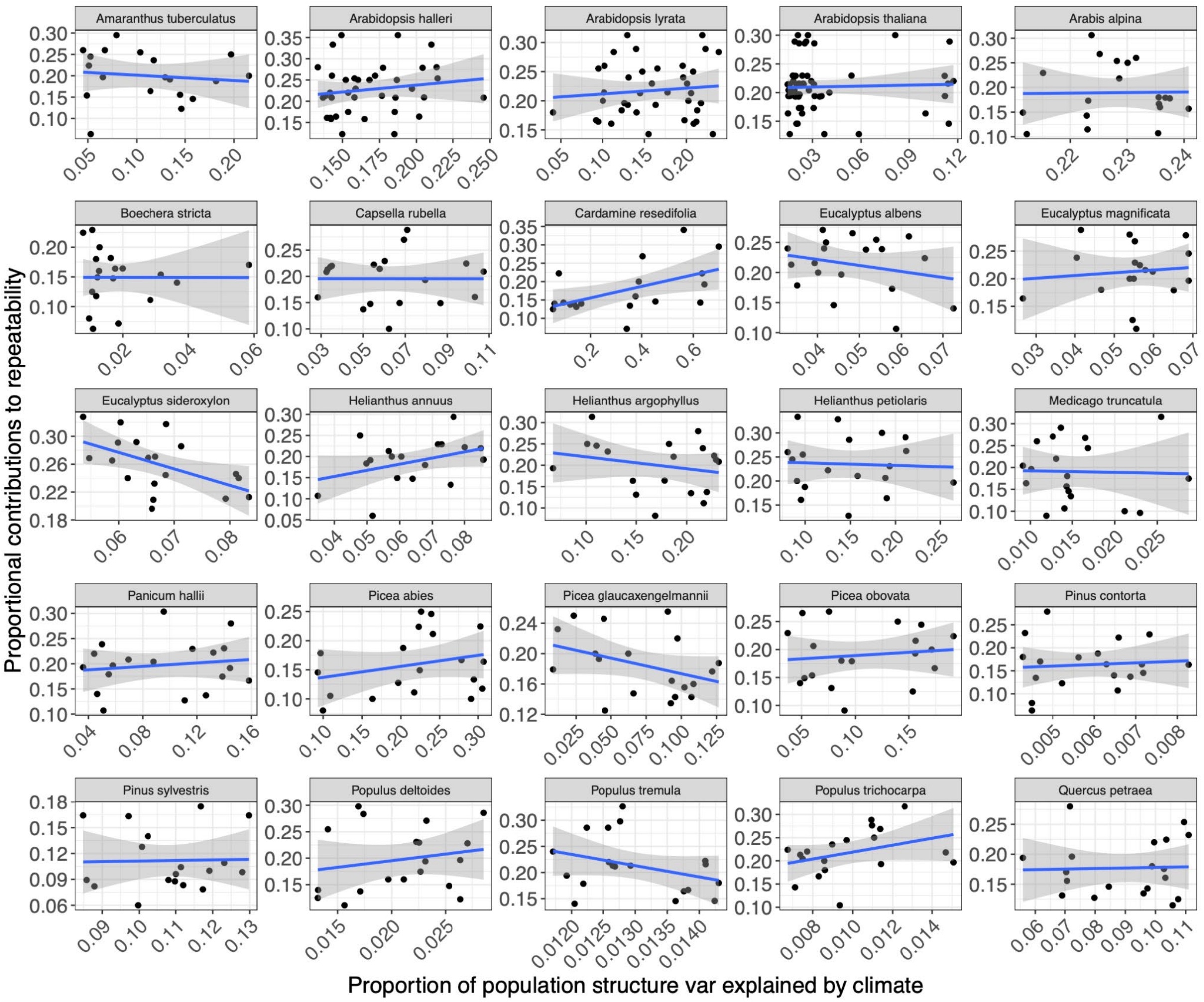

Supplementary Figure 2: *Associations between GEA power, estimated as in the inverse of how much neutral genetic variation is explained by climatic variation, and the extent to which an individual species contributes low p-values to RAOs. The y-axis represents the proportion of RAOs that include a low p-value (<0.1) from a given species. The x-axis is the proportion of neutral genetic variation that is explained by climatic variation, as estimated by pRDA. Each point therefore represents a single GEA combination of species and climate variable. Linear regression lines and standard error are included to provide a general approximation of the relationship.*

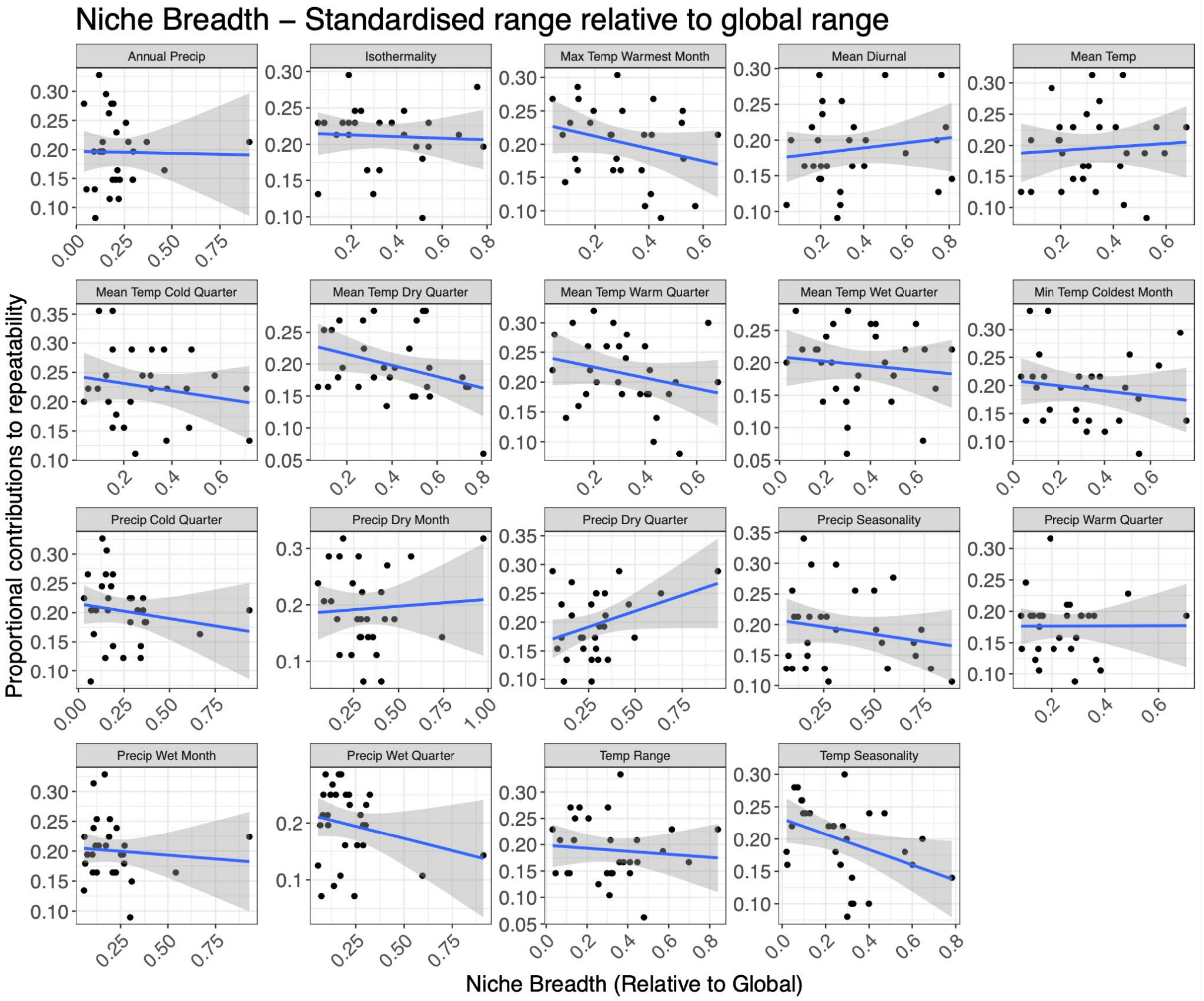

Supplementary Figure 3: *Associations between Niche Breadth, standardised by the global climatic range across all species, and the extent to which an individual species contributes low p-values to RAOs. The y-axis represents the proportion of RAOs that include a contributing p-value from a given species. Each point therefore represents a single GEA combination of species and climate variable. Linear regression lines and standard error are included to provide a general approximation of the relationship.*

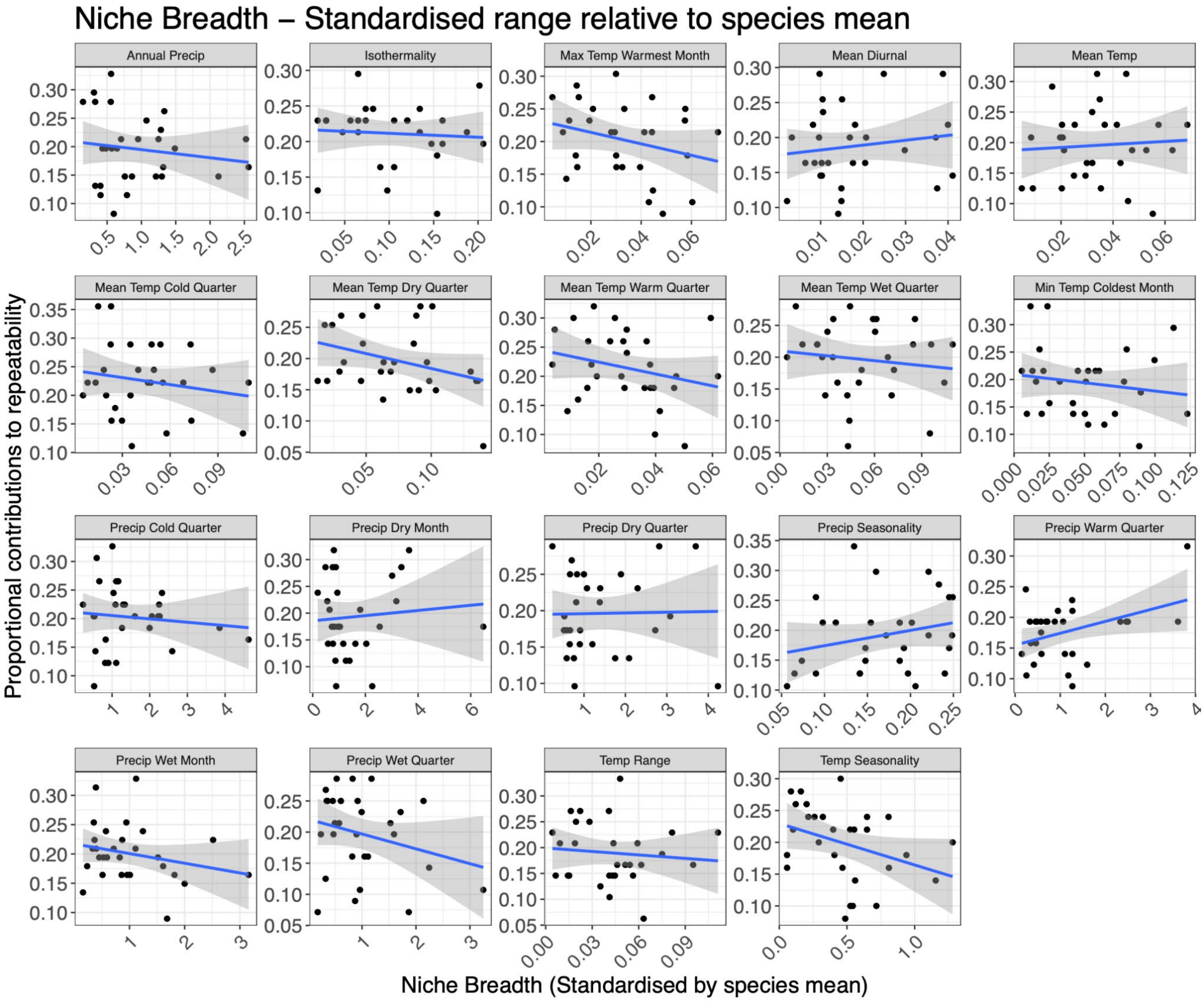

Supplementary Figure 4: *Associations between Niche Breadth, standardised by the local species mean climate, and the extent to which an individual species contributes low p-values to RAOs. The y-axis represents the proportion of RAOs that include a contributing p-value from a given species. Each point therefore represents a single GEA combination of species and climate variable. Linear regression lines and standard error are included to provide a general approximation of the relationship.*

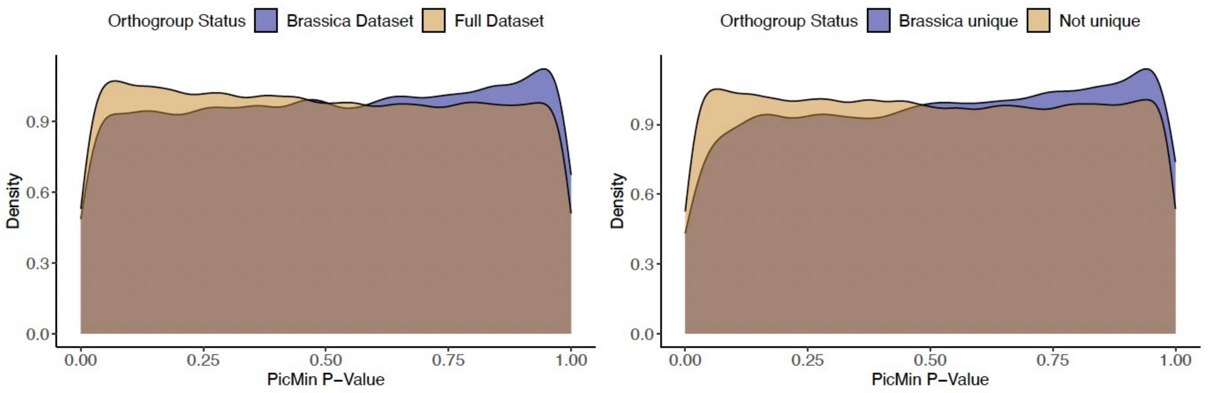

Supplementary Figure 5: *Distributions of PicMin p-values for tests of repeatability within seven Brassicaceae species. In each panel, distributions are coloured according to the status of Orthogroups in terms of whether they were tested in the main analysis (left panel) or were orthogroups that were unique to Brassicaceae (right panel). Both panels show the same result, which implies that the orthogroups with higher degrees of conservation across species, that were tested in our main analysis, also exhibit increased evidence for repeatability when tested only within Brassicaceae.*

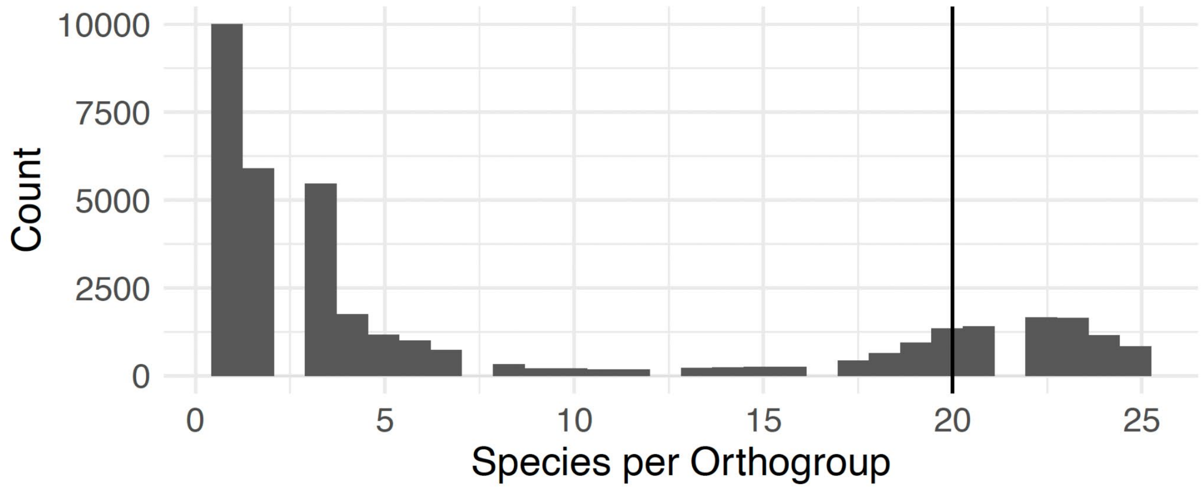

Supplementary Figure 6: *Orthogroup occupancy across species. Histogram shows the distribution of the number of species represented per orthogroup. The black line denotes the cut-off of 20 used for repeatability analyses.*

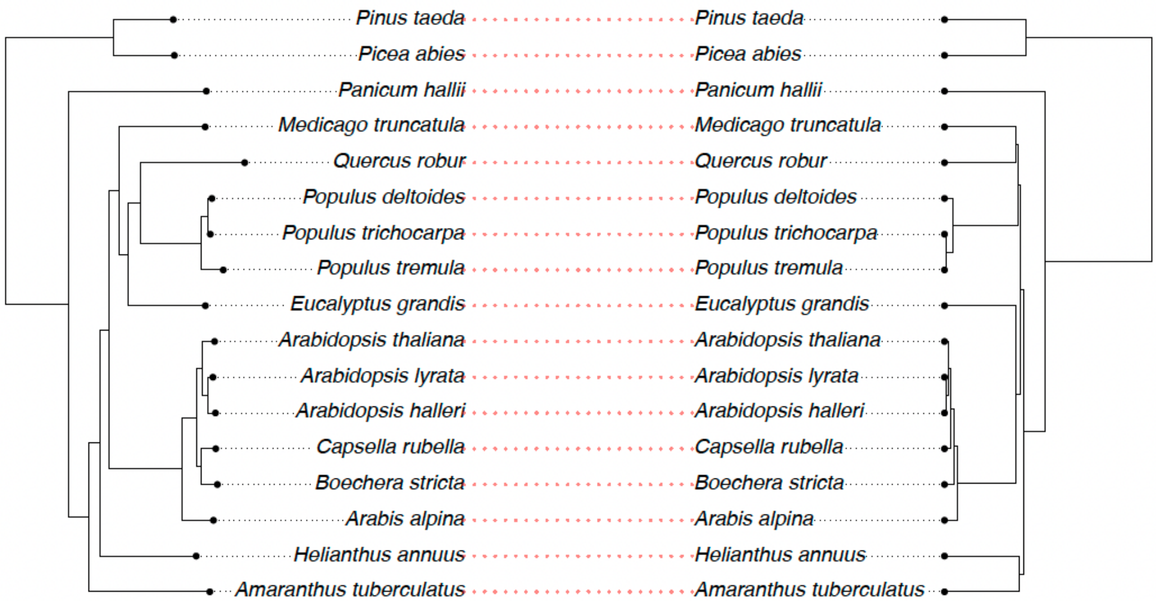

Supplementary Figure 7: *Agreement between the species-tree derived here from 5,003 orthogroups from the 17 reference genome including in this study (left), and the species tree described by TimeTree (right), which is shown in Extended Data 1.*

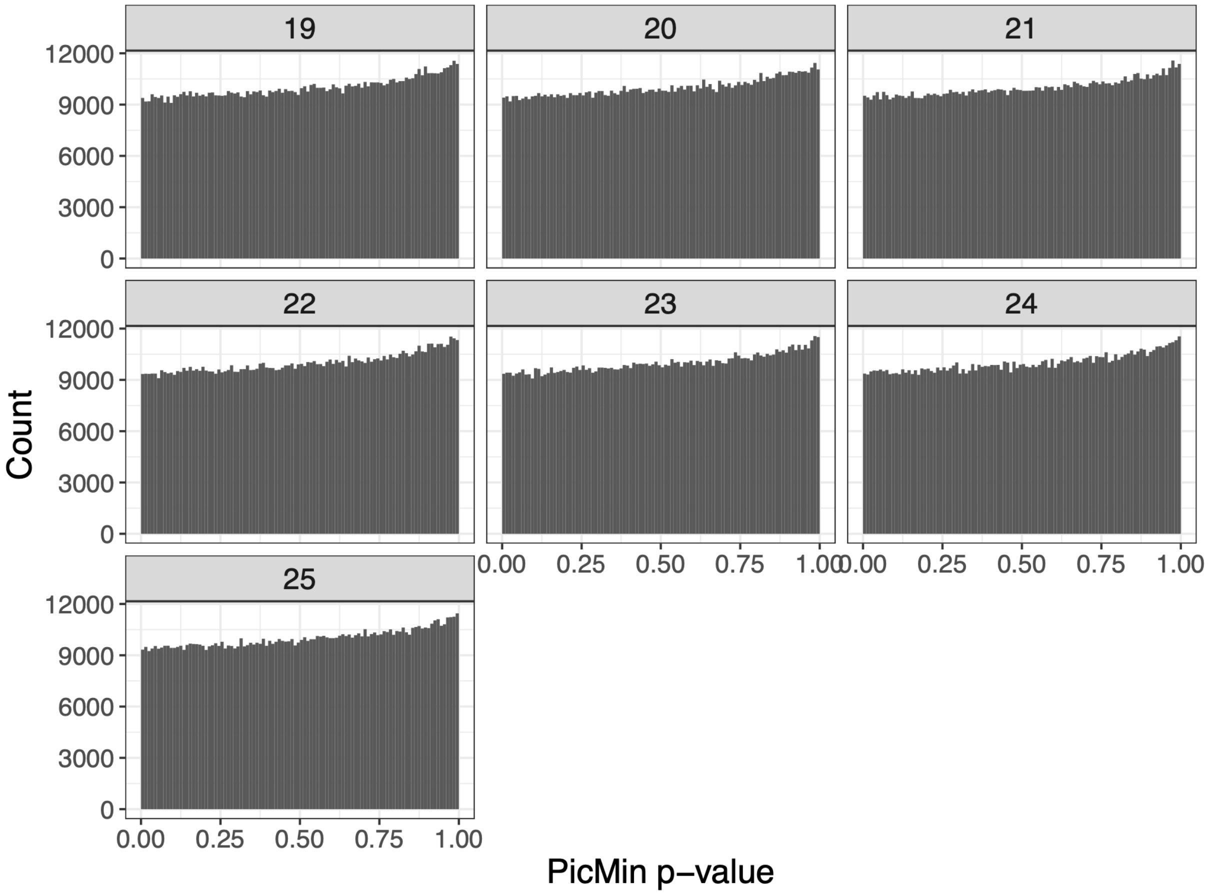

Supplementary Figure 8: *The distribution of p-values derived from PicMin under the null model that no species exhibits adaptation within a given orthogroup. Each panel shows the null distribution under a different number of tested species (19-25). These distributions were produced over 1,000,000 orthogroups per number of species derived from random uniform ep-values, and were used as an empirical null distribution to adjust final PicMin p-values.*

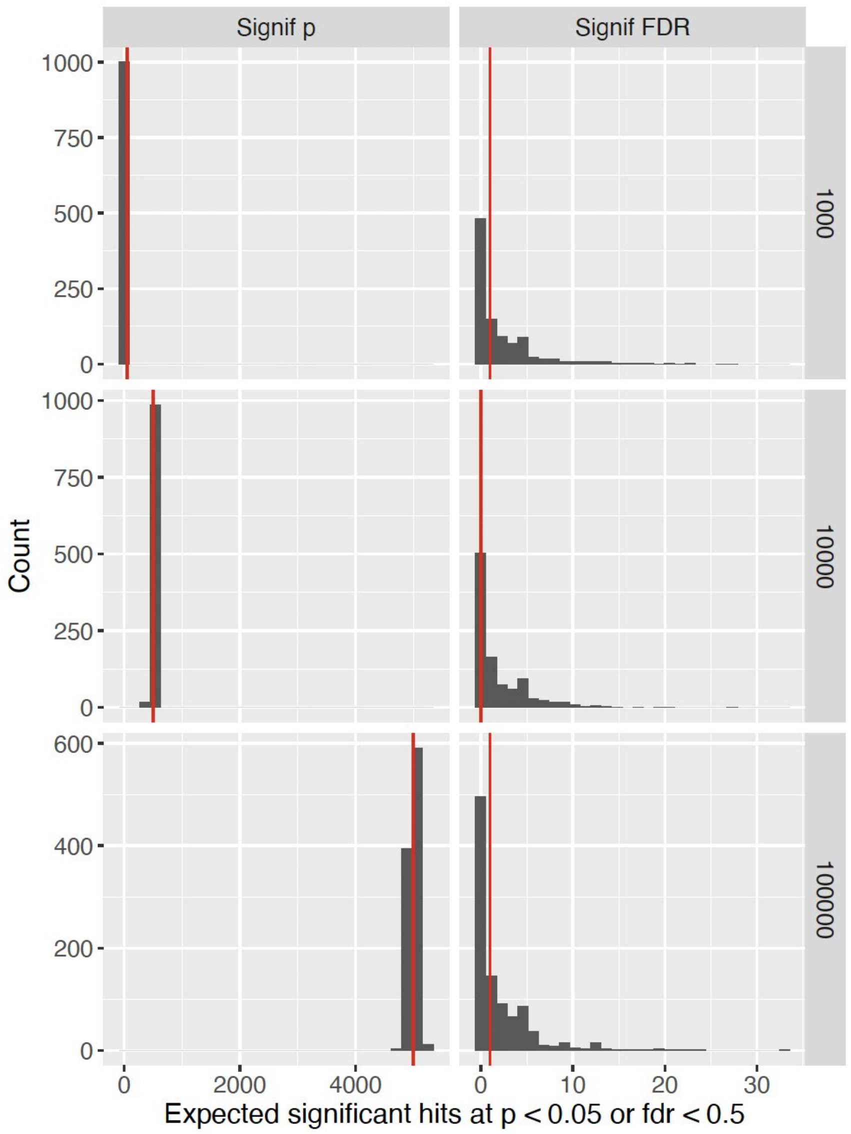

Supplementary Figure 9*: Demonstration of the different expectations for p-values and FDR-corrected q-values when selecting a cut-off. Each histogram represents 1,000 random uniform draws of p-values where all null hypotheses are true, for either 1,000, 10,000, or 100,000 tests. The 'Signif p' column shows the distribution of the number of tests with a p-value < 0.05, with the median shown as a red line. The expected number of tests with a p-value < 0.05 increases with the number of tests performed (approximately 5% of tests). The 'Signif FDR' column shows the same data FDR-corrected, with the number of tests with an FDR-corrected q-value < 0.5 (the threshold used here), with the median (either 0 or 1) shown as a red line. This simulated data demonstrates that, regardless of the number of tests being performed, there is no expectation for many tests to fall below the FDR < 0.5 threshold as there is for uncorrected p-values.*

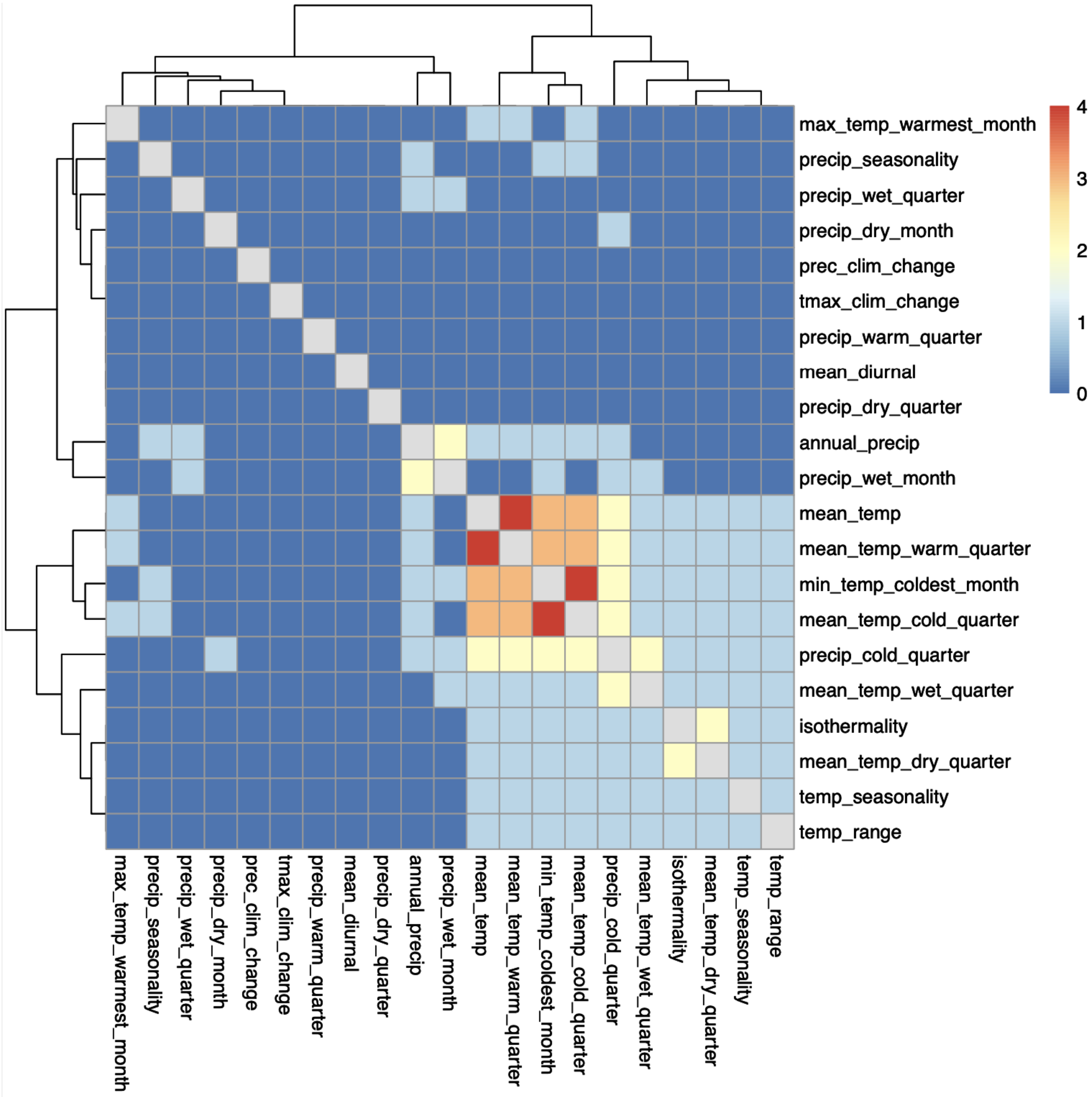

Supplementary Figure 10: *Overlap of RAOs (FDR < 0.5) among different climate variables. Each cell shows the number of orthogroups that were commonly identified through GEA associated with different climate variables. Axes are clustered with dendrograms denoting groups of climate variables with the most similar sets of orthogroups identified.*

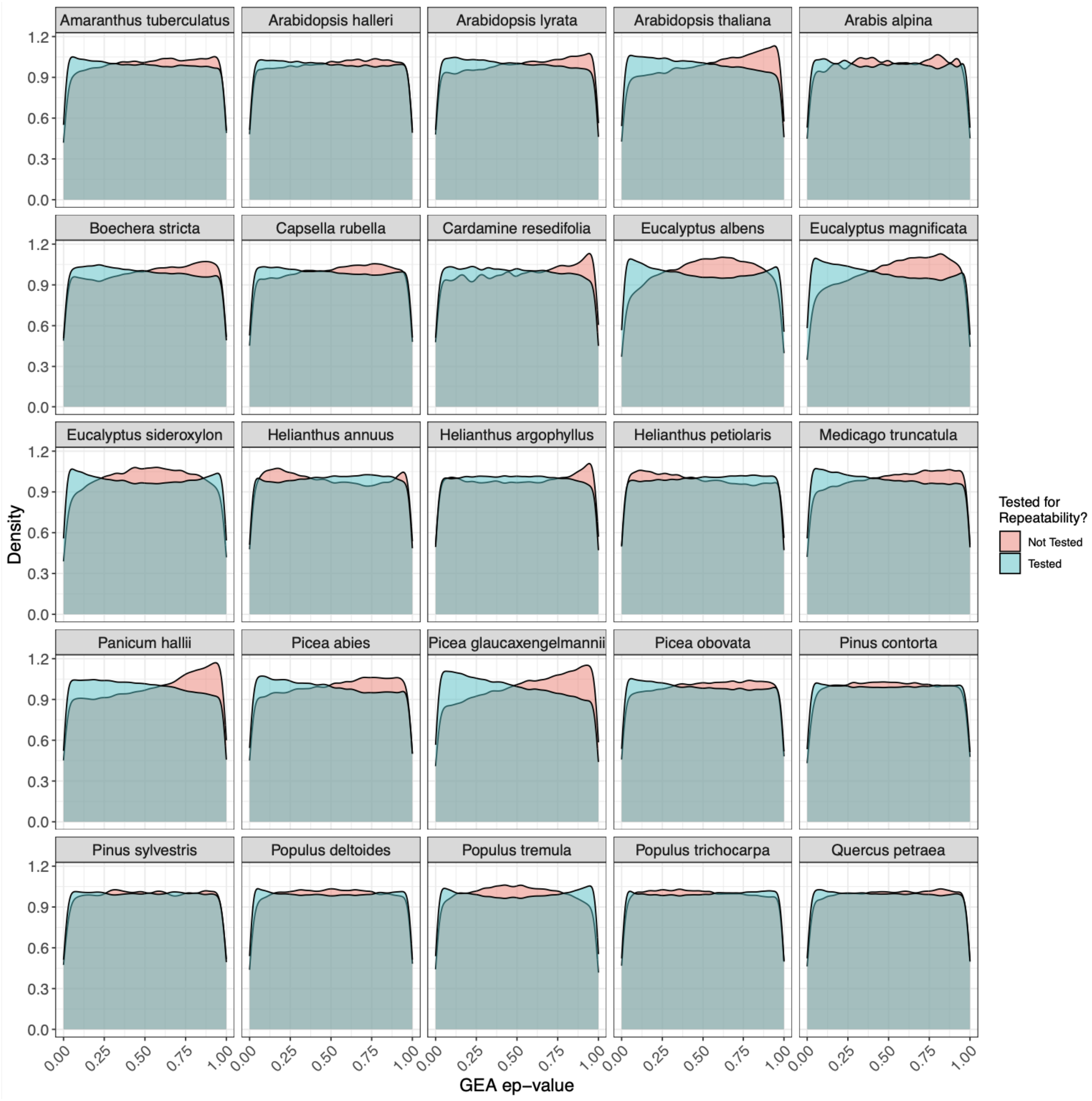

***Supplementary Figure 11**: Density distributions of tested vs not-tested orthogroup GEA ep-values. Each facet shows a different species, and fill denotes whether orthogroups were tested for repeatability or not.*

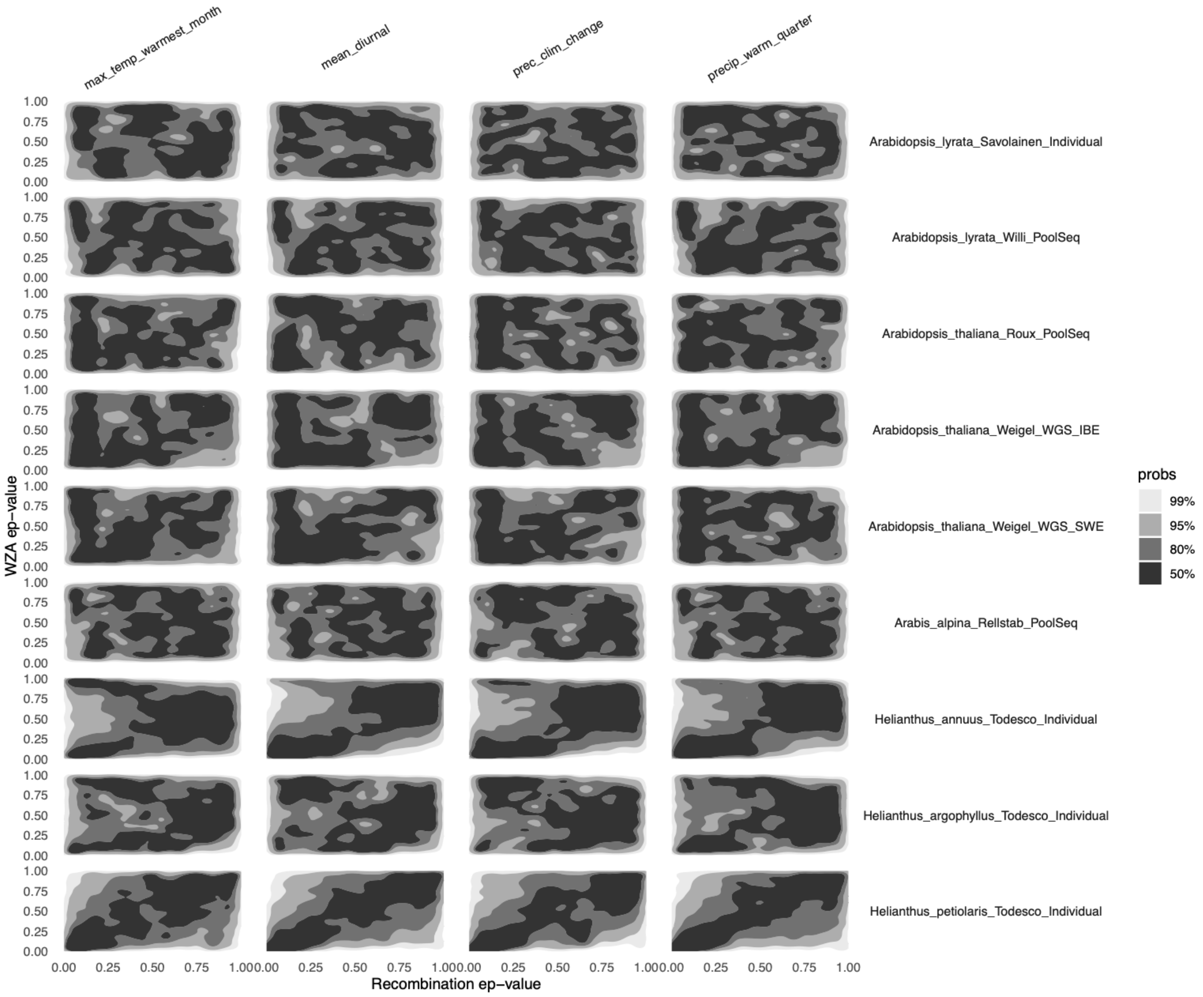

***Supplementary Figure 12**: Associations between GEA results and recombination rate. Each facet shows the density of per gene estimates of association with environment (WZA ep-value corrected for SNP count) and recombination rate (weighted mean, lower ep-value reflects lower recombination). Each axis represents a uniform distribution of empirical p-values, such that if no association exists, regions of high density are observed across the plotting area. Darker regions represent a greater density of genes.*

REFERENCES

1. Wang, M. *et al.* Phylogenomics of the genus Populus reveals extensive interspecific gene flow and balancing selection. *New Phytol.* **225**, 1370–1382 (2020).
2. Nikolov, L. A. *et al.* Resolving the backbone of the Brassicaceae phylogeny for investigating trait diversity. *New Phytol.* **222**, 1638–1651 (2019).
3. Gautier, M. Genome-Wide Scan for Adaptive Divergence and Association with Population-Specific Covariates. *Genetics* **201**, 1555–1579 (2015).
4. Luu, K., Bazin, E. & Blum, M. G. B. pcadapt: an R package to perform genome scans for selection based on principal component analysis. *Mol. Ecol. Resour.* **17**, 67–77 (2017).
5. Caye, K., Jumentier, B., Lepeule, J. & François, O. LFMM 2: Fast and Accurate Inference of Gene-Environment Associations in Genome-Wide Studies. *Mol. Biol. Evol.* **36**, 852–860 (2019).
6. Capblancq, T. & Forester, B. R. Redundancy Analysis (RDA): a Swiss Army knife for landscape genomics. *Methods Ecol. Evol.* (2021) doi:10.1111/2041-210x.13722.
7. Lotterhos, K. E. The Effect of Neutral Recombination Variation on Genome Scans for Selection. *G3* **9**, 1851–1867 (2019).
8. Lasky, J. R., Josephs, E. B. & Morris, G. P. Genotype-environment associations to reveal the molecular basis of environmental adaptation. *Plant Cell* (2022) doi:10.1093/plcell/koac267.
9. DeRaad, D. A. *et al.* Genome-environment association methods comparison supports omnigenic adaptation to ecological niche in malaria vector mosquitoes. *Mol. Ecol.* (2021) doi:10.1111/mec.16094.
10. Lasky, J. R. *et al.* Genome-environment associations in sorghum landraces predict

adaptive traits. *Sci Adv* **1**, e1400218 (2015).

11. Booker, T. R., Yeaman, S., Whiting, J. R. & Whitlock, M. C. The WZA: A window-based method for characterizing genotype-environment association. *Mol. Ecol. Resour.* (2023) doi:10.1111/1755-0998.13768.
12. Lotterhos, K. E. The paradox of adaptive trait clines with nonclinal patterns in the underlying genes. *Proc. Natl. Acad. Sci. U. S. A.* **120**, e2220313120 (2023).
13. Schlichta, F., Peischl, S. & Excoffier, L. The Impact of Genetic Surfing on Neutral Genomic Diversity. *Mol. Biol. Evol.* **39**, (2022).
14. Karunarathne, P., Zhou, Q., Lascoux, M. & Milesi, P. Hybridization mediated range expansion and climate change resilience in two conifers. *bioRxiv* 2023.01.31.526517 (2023) doi:10.1101/2023.01.31.526517.
15. Birchler, J. A. & Yang, H. The multiple fates of gene duplications: Deletion, hypofunctionalization, subfunctionalization, neofunctionalization, dosage balance constraints, and neutral variation. *Plant Cell* **34**, 2466–2474 (2022).
16. Revell, L. J. phytools: an R package for phylogenetic comparative biology (and other things). *Methods Ecol. Evol.* **3**, 217–223 (2012).
17. Blomberg, S. P., Garland, T., Jr & Ives, A. R. Testing for phylogenetic signal in comparative data: behavioral traits are more labile. *Evolution* **57**, 717–745 (2003).
18. Bohutínská, M. & Peichel, C. L. Divergence time shapes gene reuse during repeated adaptation. *Trends Ecol. Evol.* (2023) doi:10.1016/j.tree.2023.11.007.
19. Junker, R. R., Kuppler, J., Bathke, A. C., Schreyer, M. L. & Trutschnig, W. *Dynamic range boxes* – a robust nonparametric approach to quantify size and overlap of *n*-dimensional hypervolumes. *Methods Ecol. Evol.* **7**, 1503–1513 (2016).
20. Yeaman, S. *et al.* Convergent local adaptation to climate in distantly related conifers.

Science **353**, 1431–1433 (2016).

21. Guillaume, F. & Otto, S. P. Gene functional trade-offs and the evolution of pleiotropy. *Genetics* **192**, 1389–1409 (2012).
22. Booker, T. R., Yeaman, S. & Whitlock, M. C. Using genome scans to identify genes used repeatedly for adaptation. *Evolution* (2022) doi:10.1093/evolut/qpac063.
23. Jiao, W.-B. *et al.* Improving and correcting the contiguity of long-read genome assemblies of three plant species using optical mapping and chromosome conformation capture data. *Genome Res.* **27**, 778–786 (2017).
24. Hämälä, T., Mattila, T. M., Leinonen, P. H., Kuittinen, H. & Savolainen, O. Role of seed germination in adaptation and reproductive isolation in Arabidopsis lyrata. *Mol. Ecol.* **26**, 3484–3496 (2017).
25. Rowan, B. A. *et al.* An Ultra High-Density Arabidopsis thaliana Crossover Map That Refines the Influences of Structural Variation and Epigenetic Features. *Genetics* **213**, 771–787 (2019).
26. Huang, K. *et al.* Mutation Load in Sunflower Inversions Is Negatively Correlated with Inversion Heterozygosity. *Mol. Biol. Evol.* **39**, msac101 (2022).
27. Todesco, M. *et al.* Massive haplotypes underlie ecotypic differentiation in sunflowers. *Nature* **584**, 602–607 (2020).
